# Supplementary material for: Learning curve and surgical outcome of robotic assisted colorectal surgery with ERAS program
Source: Sci Rep. 2022 Nov 29;12:20566. doi: 10.1038/s41598-022-24665-w (PMC9709162; doi:10.1038/s41598-022-24665-w)
Supplement: Supplementary file 1 — Supplementary Information 1. [file 41598_2022_24665_MOESM1_ESM.zip › LSC Raw data-Ñ[▒K/053/OP_ñw╝╨░O▒Kñσ.pdf]

正式摘要(電子簽章：000000180248801)

請選擇...reporter

臺中榮民總醫院

Operation Note

[redacted]

- 手術日期：2019/11/06
- 手術醫師：VS 林俊余，CR 林昌霖，CR 江世偉，R 林陳彥
- Anesthesia : GE

Diagnosis

- Pre-operative diagnosis : T colon ca s/p loop T. Ext RH D3.
- Post-operative diagnosis : Ditto.

Surgical Indication

- For curative resection

Operation

- 1. Laparoscopic right hemicolectomy with side-to-side anastomosis, GIA 80/3.8 *2, re-inforcement with silk (+).
  2. Vessel ligation : ileo-colic, right colic, middle colic vessels and left colic artery.
  3. Cut margin: proximal (ileum) 5 cm, (colon) 30 cm, distal 15 cm.
  4. Repair of mesentery defect: nil.
  5. Laparoscopic cholecystectomy
  6. Take down the T-colostomy
  7. Two 12mm penrose drain at Morrison's pouch and splenic fossa.

Finding

- 1. A 2x1.5cm Borrmann type II ulcerative tumor at middle ascending colon, which invaded through muscular layer, but didn't penetrate through serosa. No enlarged lymph nodes at mesocolon. cT3N0M0.
  2. No other gross tumor or polyp was found.
  3. There was no visible liver metastasis or visible peritoneal seeding.
  4. Some adhesion between A-colon and T-colon.

Procedure

- General endotracheal anesthesia was induced and then the patient was placed in the supine position with legs in adducted position. Preoperative antibiotics were given. A Foley catheter and nasogastric tube were placed. The abdomen was prepped and draped in standard sterile fashion. A small vertical incision was made below the umbilicus and a 12mm trocar was inserted into the peritoneal cavity by open method. Pneumoperitoneum was established with CO2 inflation. Laparoscope was inserted and then a 12mm trocar were inserted over LUQ of abdomen. Two 5mm trocars were inserted over RUQ and LLQ of abdomen. The liver, omentum and peritoneum were inspected for evidence of metastatic disease. No metastatic disease was noted. laparoscopic cholecystectomy was done by GS doctor.The small bowel was inspected and retracted to downward by grasper. The peritoneum overlying the mesentery was then scored with electrocautery and the ileocolic artery was identified, ligated with endoclips, and transected. The remaining vessels to the cecum were also divided and ligated. Using electrocautery, omentum was freed from the transverse colon and lesser sac was entered. The colon was freed from its peritoneal attachments along the line of Toldt from the cecum to the hepatic flexure. CO2 inflation was discontinued and T colon stoma was took down for better operatrive field, the stoma wound was protected with wound retractor. Using electrocautery, splenic flexure was took down and ligated left colic artery. Hemostasis was checked in the operative field. Cecum, ascending colon,hepatic flexure, transverse colon and splenic flexure were mobilized and then laparoscope was removed. The rected colon was delivered into extraperitoneaum, the proximal and distal end was made with side-to-side

- anastomosis with GIA 80/3.8. Interrupted 3-0 silk Lambert sutures were used to re-inforcement at outer layer. The anastomosis was checked and found to be intact and widely patent. Two 12mm penrose drain were placed at sub-hepatic region and splenic fossa. The fascia was closed with interrupted sutures of No.1 Vicryl. The fascia of the 12 mm trochar wound was closed with 1-0 Vicryl. The skin was approximated with 3-0 Nylon. The patient tolerated the procedure well and was brought to the POR in stable condition
- Estimated blood loss: 450 c.c
- 器械數量: 正確
- 紗布數量: 正確

Image

- PAINT
  - abdomen

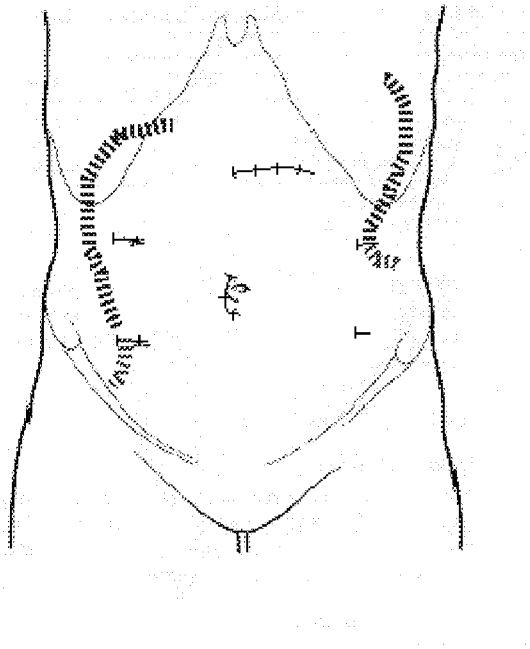

  - colon & rectum

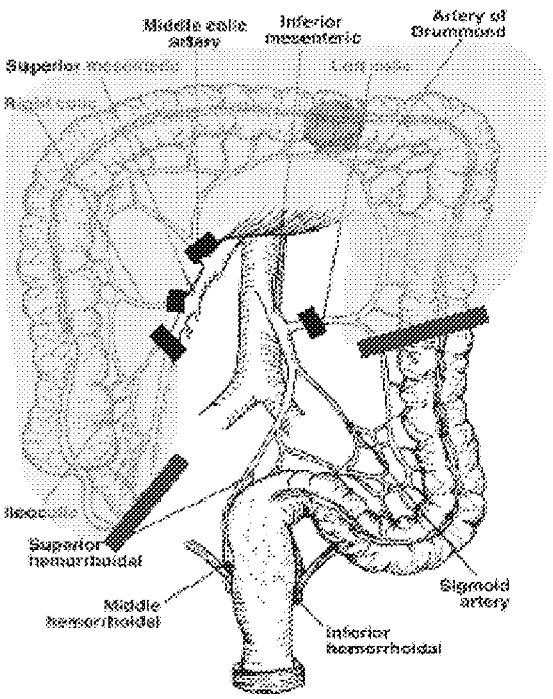

編輯主治 2222H林俊余、主治 2288K江世偉

正式摘要**(電子簽章：000000180248801)**

請選擇...reporter

doctorRank:B
